# Two-terminal floating-gate memory with van der Waals heterostructures for ultrahigh on/off ratio

**DOI:** 10.1038/ncomms12725

**Published:** 2016-09-02

**Authors:** Quoc An Vu, Yong Seon Shin, Young Rae Kim, Van Luan Nguyen, Won Tae Kang, Hyun Kim, Dinh Hoa Luong, Il Min Lee, Kiyoung Lee, Dong-Su Ko, Jinseong Heo, Seongjun Park, Young Hee Lee, Woo Jong Yu

**Affiliations:** 1 Center for Integrated Nanostructure Physics, Institute for Basic Science (IBS), Suwon 16419, Republic of Korea; 2 Department of Energy Science, Sungkyunkwan University, Suwon 16419, Republic of Korea; 3 Department of Electronic and Electrical Engineering, Sungkyunkwan University, Suwon 16419, Republic of Korea; 4 Samsung Advanced Institute of Technology, Suwon 16678, Republic of Korea; 5 Samsung-SKKU Graphene Center (SSGC), Sungkyunkwan University, Suwon 16419, Republic of Korea

## Abstract

Concepts of non-volatile memory to replace conventional flash memory have suffered from low material reliability and high off-state current, and the use of a thick, rigid blocking oxide layer in flash memory further restricts vertical scale-up. Here, we report a two-terminal floating gate memory, tunnelling random access memory fabricated by a monolayer MoS_2_/h-BN/monolayer graphene vertical stack. Our device uses a two-terminal electrode for current flow in the MoS_2_ channel and simultaneously for charging and discharging the graphene floating gate through the h-BN tunnelling barrier. By effective charge tunnelling through crystalline h-BN layer and storing charges in graphene layer, our memory device demonstrates an ultimately low off-state current of 10^−14^ A, leading to ultrahigh on/off ratio over 10^9^, about ∼10^3^ times higher than other two-terminal memories. Furthermore, the absence of thick, rigid blocking oxides enables high stretchability (>19%) which is useful for soft electronics.

## Abstract (web summary)

Traditional non-volatile memories suffer from poor scalability in the vertical direction due to the use of a bulky oxide layer. Here, the authors develop a tunnelling random access memory using a vertical heterostructure composed of atomically thin molybdenum disulfide, boron nitride and graphene.

## Graphical abstract

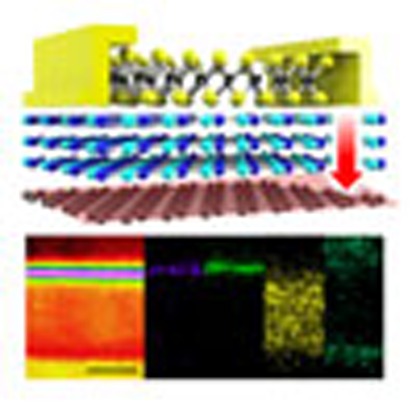

Three-terminal device (source, drain and gate) of flash memory^1^ increases circuit complexity and requires long gate channel length which limits integration density. Furthermore, the presence of a thick and rigid blocking oxide layer leads to high power consumption^2^,^3^ and restricts scaling up in the vertical direction, and limits the flexibility and stretchability of the devices for applications^4^,^5^. Two-terminal memory devices have been proposed to overcome such limitations: Phase-change random-access memory (PRAM)^6^,^7^,^8^ and resistive random-access memory (RRAM)^2^,^9^,^10^. Due to the absence of gate electrode, two-terminal memories can have extremely short channel length to achieve high memory integration. Furthermore, two-terminal memories can behave as memristor^9^ that have potential for applications of semi-non-volatile memories and learning networks that require a synapse-like function^9^,^10^. However, the biggest challenge for two-terminal memories are low reliability and high leakage off-current because of thermally activated hopping transport through trap states (Poole–Frenkel conduction)^11^,^12^,^13^,^14^,^15^, resulting in low on/off ratio and off-state power consumption.

Meanwhile, van der Waals heterostructures based on two-dimensional (2D) materials such as graphene, semiconducting transition metal dichalcogenides and insulating hexagonal boron nitride (h-BN) exhibit peculiar electronic^16^,^17^,^18^,^19^,^20^ and optoelectronic properties^21^,^22^,^23^,^24^,^25^, and furthermore can provide transparency, flexibility and stretchability^19^,^21^,^23^,^26^,^27^. The h-BN layer, with a large bandgap of 5.97 eV, is used as a high-quality insulator^20^,^28^ and tunnelling barrier^17^,^24^.

Here, we propose a two-terminal tunnelling random access memory (TRAM) using a vertically stacked MoS_2_/h-BN/Gr heterostructure. With appropriate thickness of h-BN, charges can tunnel through h-BN layer and be stored in the floating gate by large potential difference between drain and floating gate, while the stored charges in graphene cannot tunnel out to source electrode due to the absence of potential difference, thus allowing for charge storage in the floating gate. Our TRAM demonstrates an ultralow off-state current (10^−14^ A), leading to ultimately high on/off ratio (over 10^9^). In addition, we do not use gate electrode and thick, rigid blocking insulator and instead use an ultrathin h-BN (<10 nm) with high Young's modulus and breaking strength, which is certainly advantageous for high flexibility, stretchability and scalability in the vertical direction. Our device performs excellent stretchability with no significant electrical performance degradation up to 19% strain, which will be used for wearable and body-attachable electronics in the near future.

## Results

### Device structure and memory characteristic of TRAM

**Figure 1:**
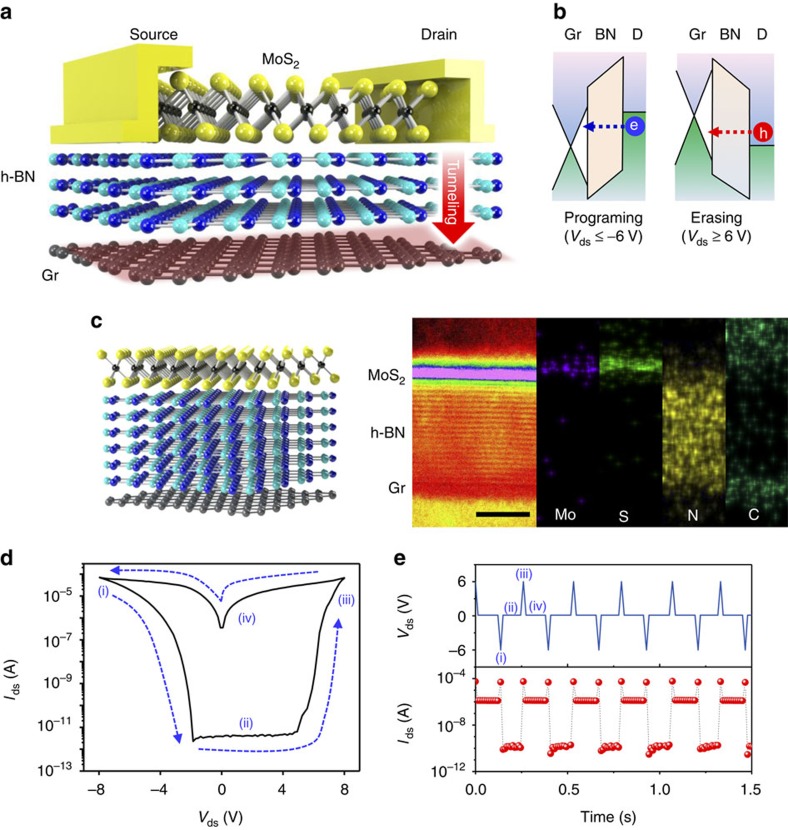
Device structure and memory characteristics of the TRAM. (**a**) Schematic of the two-terminal TRAM with monolayer MoS_2_ as a semiconducting channel at the top, h-BN as a tunnelling insulator in the middle and monolayer graphene as a floating gate, charge tunnelling between drain and graphene is shown by red arrow. (**b**) Band diagrams of drain (D)/h-BN/graphene (Gr), the dashed line arrows indicate the tunnelling direction of electrons and holes. Electrons are tunnelled from the drain to graphene at the *V*_ds_≤−6 V (Programme) and holes are tunnelled from h-BN to graphene at *V*_ds_≥6 V (Erase) states. (**c**) Atomistic schematic for the TRAM heterostructure of the monolayer MoS_2_/multilayer h-BN/monolayer graphene (left side) and cross-sectional bright-field scanning transmission electron microscope image and energy-dispersive X-ray spectroscopy elemental mapping of the TRAM heterostructure with 10-nm h-BN (right side). Scale bar is 5 nm. (**d**) Typical *I*–*V* curve of the TRAM with 5.5-nm thick h-BN. The current sweep by sweeping *V*_ds_ is shown as a dashed line. The current sweep can be separated into four stages: (i) Programme, (ii) Read, (iii) Erase and (iv) Read. Channel length and channel width of the device are 4 and 2 μm, respectively. (**e**) Repeated Erase/Read/Programme/Read sequence with a drain voltage of +6 V/+0.1 V/−6 V/+0.1 V, respectively. The pulse width was 0.01 s.

Figure 1a demonstrates the structure of TRAM with MoS_2_ on the top, h-BN in the middle and graphene at the bottom. Two Cr/Au electrodes were contacted with the monolayer MoS_2_ channel for source and drain (Supplementary Figs 1 and 2). Figure 1b shows the band diagrams of drain/h-BN/graphene in programme (*V*_ds_≤−6 V) and erase states (*V*_ds_≥6 V). The atomistic model (left side Fig. 1c) and cross-sectional bright-field scanning transmission electron microscope image show that the fabricated heterostructure is atomically flat and free from interlayer contaminants (right side Fig. 1c). Each layer was identified by energy-dispersive X-ray spectroscopy. Elements of Mo and S for MoS_2_, N for h-BN and C for graphene were observed in each stacking position.

Charging and discharging processes in the graphene are conducted by controlling drain voltage (Fig. 1d), well distinguished from conventional floating gate memory (CFGM) that operates by the gate voltage with three terminals (Supplementary Fig. 3)^29^,^30^. For a voltage sweep from 0 to ±8 V (sweeping directions indicated by the arrow in Fig. 1d), the resistivity of MoS_2_ changes from high-resistance (OFF) state to low-resistance (ON) state and back to OFF state, yielding a current hysteresis. This hysteresis behaviour is reproducible during the subsequent series of voltage sweeps. Figure 1e demonstrates a series of memory cycles using the repeated voltage pulses of −6, 0.1, 6 and 0.1 V as programing (i), reading (ii), erasing (iii) and reading (iv) operations, respectively. A non-destructive read state with an ON/OFF ratio over 10^4^ was achieved, which can also be inferred from the current hysteresis loop shown in Fig. 1d. To ensure the hysteresis of our devices originating from charging in graphene and the real current flows through MoS_2_ channel, graphene was grounded and no hysteresis was observed (Supplementary Fig. 4).

### Proposed operation principle of TRAM

**Figure 2:**
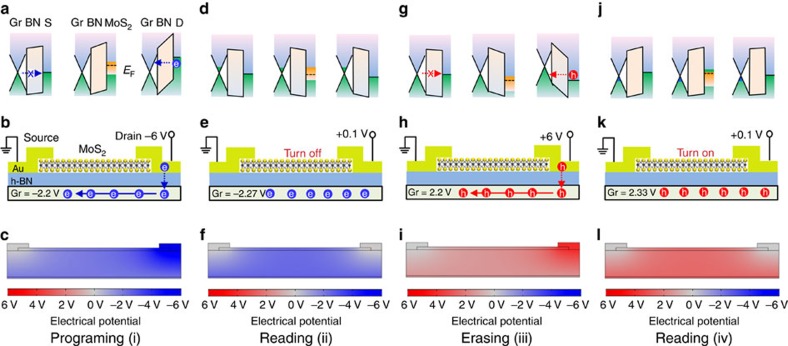
Numerical simulation of potential distribution in TRAM and the schematic band diagram. Schematic illustration of band diagram, memory operation and simulated electrostatic potential at the states of Programme (**a**–**c**), Read in Programme state (**d**–**f**), Erase (**g**–**i**) and Read in Erase state (**j**–**l**). The dashed line arrows indicate the tunnelling direction of electrons and holes from drain into graphene. The solid line arrows indicate the spreading of electrons and holes at graphene after tunnelling.

Such high TRAM performance originates from the asymmetric potential drop built in two contacts between source/floating gate and drain/floating. Figure 2a–c shows the schematic band diagrams (Fig. 2a), charge tunnelling (Fig. 2b), and the simulated potential profile (Fig. 2c) in the programme states, read in off-state (Fig. 2d–f), erase (Fig. 2g–i), and read in on-state (Fig. 2j–l), corresponding to the respective i, ii, iii and iv stages in Fig. 1d,e. The geometry and parameters of the simulation model are shown and discussed in Supplementary Note 1, Supplementary Figs 5 and 6 and Supplementary Tables 1 and 2. Typical channel length of our device is about 2 μm. In fact, we performed two simulations for two channel lengths: 30 nm and 2 μm. The potential distributions in both channel lengths were almost similar to each other (Supplementary Fig. 7). Therefore, we used the simulation model with the channel length of 30 nm for better eye capturing. In the electrostatic potential simulation of the programme (bottom panel of Fig. 2a), the large potential difference and the corresponding large electric field were constructed between drain and graphene, whereas the negligible potential difference and the corresponding low electric field (Supplementary Fig. 8a) were exhibited between source and graphene. A large potential drop between the graphene and drain by −6 V drain bias enables electron tunnelling from drain to graphene, and electrons are spread out through the entire graphene (Fig. 2b). Meanwhile, electrons in graphene cannot tunnel out to MoS_2_ or source because of the small potential drop at the contact (Fig. 2c). The asymmetric potential drop originates from the highly resistive MoS_2_ that competes with tunnelling probability through the thin h-BN film, which is a key concept to store charges in graphene.

The reading process of the programme state is shown in Fig. 2d–f. Because the trapped electrons in the programme state charge the graphene, the graphene potential could be dropped to maintain −2.27 V (Supplementary Note 1). The small potential difference (Fig. 2f) and the corresponding low electric field (Supplementary Fig. 8b) are shown at both the drain–graphene and source–graphene junctions, which are low enough to prevent leakage of the trapped electrons in the floating gate. Meanwhile, the trapped electrons generate a negative electric field and deplete the majority electron carriers in the MoS_2_, resulting in a highly resistive state. Consequently, the MoS_2_ performs the programme state (off-state). At +6 V, drain bias (Fig. 2g–i and Supplementary Fig. 8c) holes are tunnelled from drain and trapped in graphene, generating a highly conductive inversion channel in the MoS_2_ (Fig. 2j–l and Supplementary Fig. 8d), performing an erase state (on-state). Consequently, a large electrical hysteresis is formed by tunnelling and trapping electrons and holes in graphene by a simple drain-bias sweep, fundamental difference from the three-terminals flash memory CFGM (Supplementary Note 2 and Supplementary Fig. 9). By calculating carrier concentrations in graphene and MoS_2_ (Supplementary Note 3, Supplementary Fig. 10), the carrier concentration of graphene floating gate at Read in Erase state are 1.2 × 10^13^ cm^−2^, higher than that of undoped graphene. MoS_2_ channel shows a carrier concentration of 1.0 × 10^13^ cm^−2^, higher than typical carrier density of MoS_2_ channel, congruent with higher source–drain current in our device.

### The h-BN layer thickness dependence

**Figure 3:**
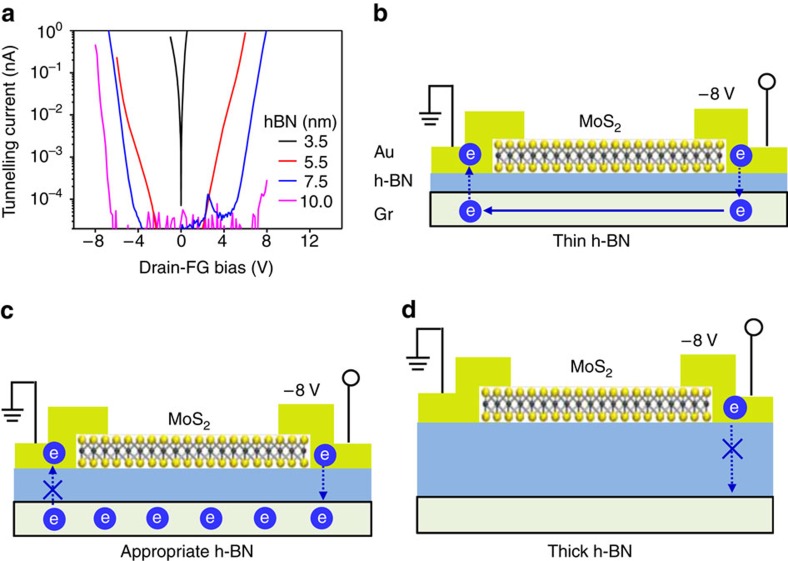
Tunnelling current through h-BN layer. (**a**) Tunnelling current characteristics between drain electrode and graphene floating gate for various h-BN thicknesses. (**b**–**d**) Schematics of electron tunnelling between electrode and floating gate at different h-BN thicknesses. A too-thin h-BN layer allows for charge tunnelling on both contacts, and no charges are stored in the graphene (**b**); a too-thick h-BN layer prohibits tunnelling current at all (**d**). An appropriate thickness of the h-BN layer is necessary to invoke the asymmetric potential drop so that charges can be stored in the graphene without appreciable leakage current (**c**).

To investigate the thickness dependence of h-BN film on tunnelling current, we fabricated TRAM devices with various h-BN thicknesses from 3.5 to 12 nm. The tunnelling threshold voltage increased in proportion to the h-BN thickness (Fig. 3a). The thin h-BN (3.5 nm) device exhibits a measurable low-bias conductance, which is attributed to direct tunnelling through the thin h-BN film. Meanwhile, the devices with 5.5, 7.5 and 10 nm thick h-BN film show the threshold voltage at ±2, ±5 and ±7 V, respectively, at a current limit of 10^−13^ A (Supplementary Fig. 11). Over the threshold voltage regime, the tunnelling current becomes nonlinear, which is attributed to Fowler–Nordheim tunnelling^31^. The tunnelling behaviour depending on the h-BN thickness largely influences charge trapping in graphene, as illustrated in Fig. 3b.

**Figure 4:**
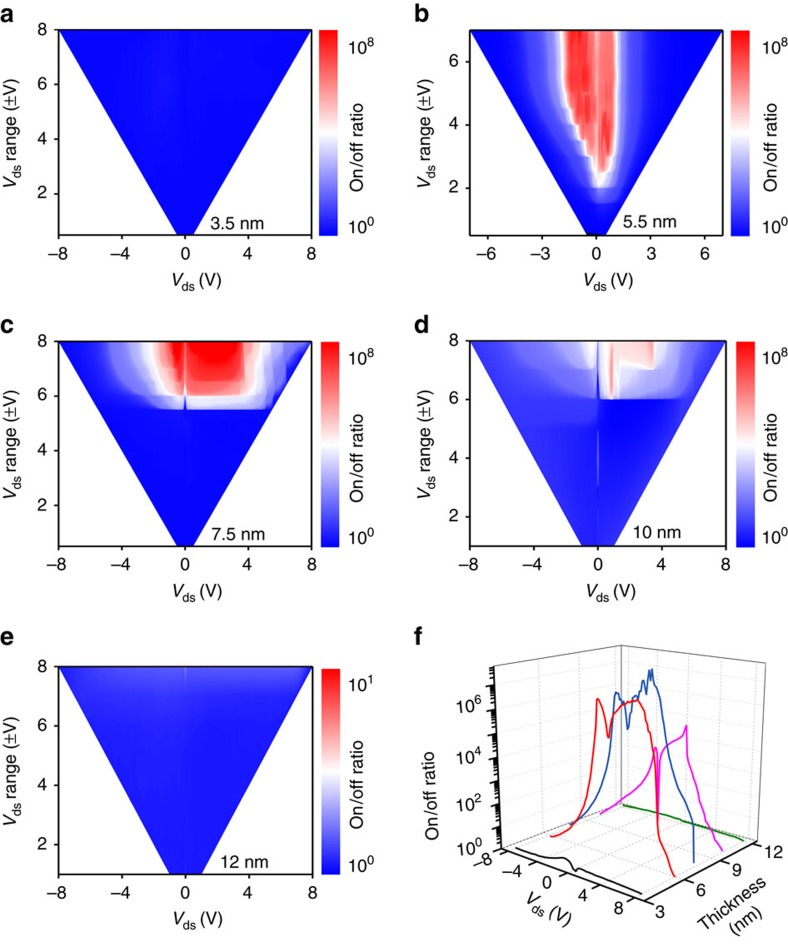
The h-BN thickness dependence of TRAM. (**a**–**e**) Colour plots of on/off ratio values as a function of source–drain voltage (*x*-axis) for a given maximum sweeping voltage (*y*-axis) with indicated h-BN thickness. (**f**) On/off ratio as functions of *V*_ds_ and thickness.

Figure 4a–e shows 2D colour images of on/off ratio for various thicknesses of h-BN layer (Supplementary Figs 12–16). For a thin h-BN (3.5 nm), on/off ratio was low in any drain sweep as discussed previously (Fig. 4a). At appropriate h-BN thicknesses of 5.5 nm (Fig. 4b) and 7.5 nm (Fig. 4c), high on/off ratio was obtained above the tunnelling threshold voltages of 2 and 5 V, respectively, and increased up to over 10^8^ (red colour area) at reasonable tunnelling threshold voltages. For a 10-nm h-BN, the threshold voltage exceeded 7 V and the obtained on/off ratio was not high because of insufficient tunnelling current via the thick h-BN insulator (Fig. 4d). For a 12-nm h-BN, the on/off ratio was ∼1 because of no tunnelling current through such a thick h-BN (Fig. 4e). Figure 4f shows a three-dimensional plot of on/off ratio in terms of drain sweep voltage and h-BN thickness. The highest on/off ratio is exhibited at an h-BN thickness ∼5.5–7.5 nm. However, a 7.5-nm device maintained a better on-current retention than a 5.5-nm h-BN device because of the reduced leakage current at thicker h-BN film (Supplementary Fig. 17).

### Memory performance of TRAM

**Figure 5:**
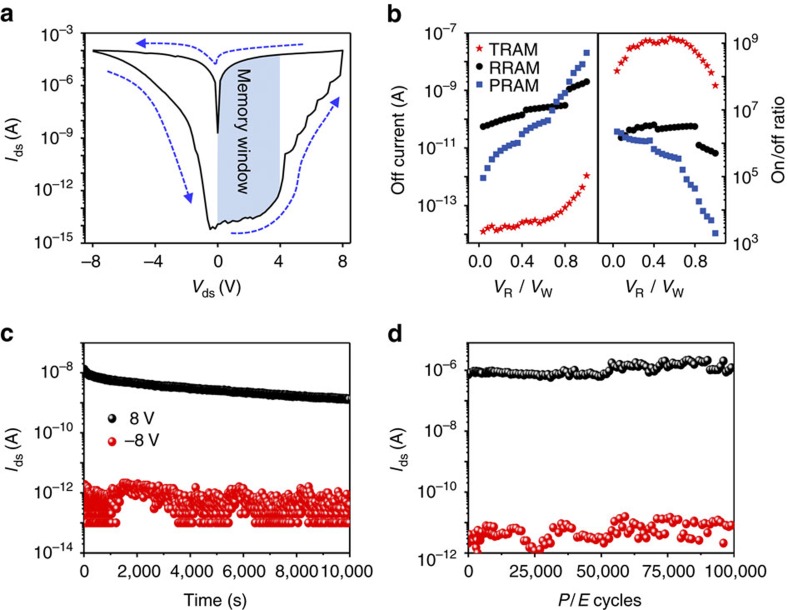
Memory performance of TRAM. (**a**) *I*–*V* curve of the TRAM with 7.5-nm thick h-BN. Channel length and channel width of the device are 4 and 2 μm, respectively. The arrows indicate the current sweeping direction. Memory window from 0 to 4 V is shown with blue colour. (**b**) The plot of off current (left panel) and on/off ratio (right panel) along with reading voltage (*V*_R_) normalized by memory window (*V*_W_). (**c**) Retention characteristics of TRAM with 7.5-nm thick h-BN after programme (*V*_ds_=−8 V) and erase (*V*_ds_= 8 V) for 5 s, *V*_reading_=0.01 V. (**d**) Endurance characteristics of the TRAM. Programme and erase were carried out by −6 V and +6 V with a pulse width of 0.1 s and a reading voltage of 0.1 V.

We further investigated the memory performance with a 7.5 nm h-BN film. Figure 5a shows a typical *I*–*V* characteristic with a sweep range of ±8 V. The memory window (*V*_W_) of our TRAM was shown at *V*_ds_=0–4 V, revealing an on-current of 10^−4^–10^−3^ A and off-current of 10^−14^ A (on/off ratio over 10^9^). The off-current and on/off ratio of TRAM (red star), RRAM^32^ (black circle) and PRAM^33^ (blue square) are plotted in Fig. 4b. Our TRAM shows 10^3^–10^4^ times lower off-state leakage current (left panel of Fig. 5b) and ∼10^3^ times higher on/off ratio (right panel of Fig. 5b) because of the effective charge tunnelling through crystalline h-BN layer and storing charges in graphene layer, which can reduce the off-state power consumption, avoid the data sensing error caused by circuit noise and allow the multi-level cell for storing more than a single bit information. P-RAM and R-RAM can be suitable for high integration due to their vertical geometry. However, small on/off ratio limits their application for multi-level memory. Our device demonstrated 100–1,000 times larger on/off ratio than P-RAM or R-RAM, which is encouraging for multi-level memory applications, as demonstrated in Supplementary Fig. 18. Arithmetically, single four-level TRAM can replace two two-level P-RAM or R-RAM. This may compensate for integration density of the planar structure.

In memory characterization, the on and off currents showed no appreciable change, while maintaining an on/off ratio of 10^4^ during a prolonged time scale (>10^4^ s) (Fig. 5c), similar to the retention characteristic of gate-bias induced CFGM (Supplementary Fig. 19). The advantages of our TRAM compared with CFGM are demonstrated in Supplementary Fig. 20 and Supplementary Note 4. In the endurance test, excellent memory durability and stability were exhibited with an on/off ratio of 10^5^ over 10^5^ cycles (Fig. 5d) by retaining a large hysteresis (Supplementary Fig. 21). In addition, our TRAM showed high reproducibility, fast programme/erase operation (<5 ms) and temperature stability over 510 K (Supplementary Figs 22–24). To confirm that our TRAM can operate without a control gate, we measured the *I*–*V* characteristics of the device with and without grounded Si. In both cases, the memory behaviour was identical (Supplementary Fig. 25). We fabricated another device that has no metal pad on graphene layer. There was no difference between with and without metal pad (Supplementary Fig. 26).

### Flexibility and stretchability

**Figure 6:**
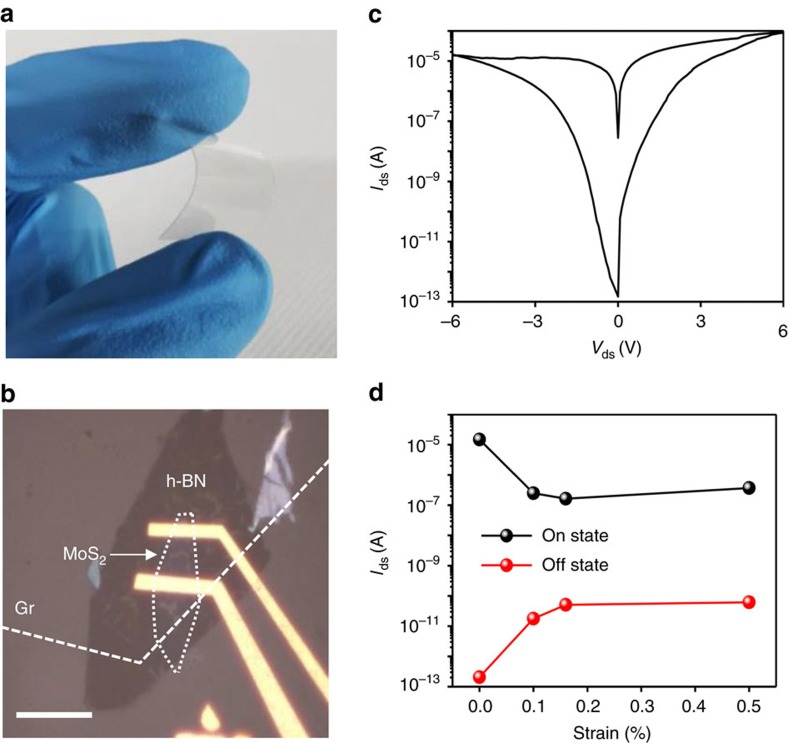
Flexibility of TRAM. (**a**–**b**) Optical images of TRAM on PET flexible substrate of a 7-nm thick h-BN layer, scale bar is 10 μm. (**c**) *I*–*V* characteristic of memory device fabricated on PET substrate. (**d**) On- and Off-current of TRAM with applying strain at a given voltage of −0.5 V.

The absence of a gate electrode and thick blocking insulator as well as an ultrathin thickness (<10 nm) using 2D materials with high Young's modulus and breaking strength^34^,^35^,^36^ is certainly advantageous for both high flexibility and stretchability. We fabricated TRAM on flexible polyethylene terephthalate (PET) substrate to investigate the flexibility (Fig. 6a,b). *I*–*V* characteristics show again the clear memory behaviour with an on/off ratio over 8 × 10^7^ at *V*_ds_=−0.5 V (Fig. 6c). This indicates that the memory behaviour originates not from the drain–Si coupling effect but from the drain–floating gate coupling effect. In the bending test, the on/off ratio was reduced from 10^8^ to 10^4^ with the applying bending strain of 0.15%, which could be attributed to poor contact between metal/channel and/or metal/h-BN/Gr; however, the ratio was maintained to 10^4^ to ∼0.5 % (maximum strain in PET bending), as expected from the strains of the used materials^34^,^35^,^36^, as shown in Fig. 6d.

**Figure 7:**
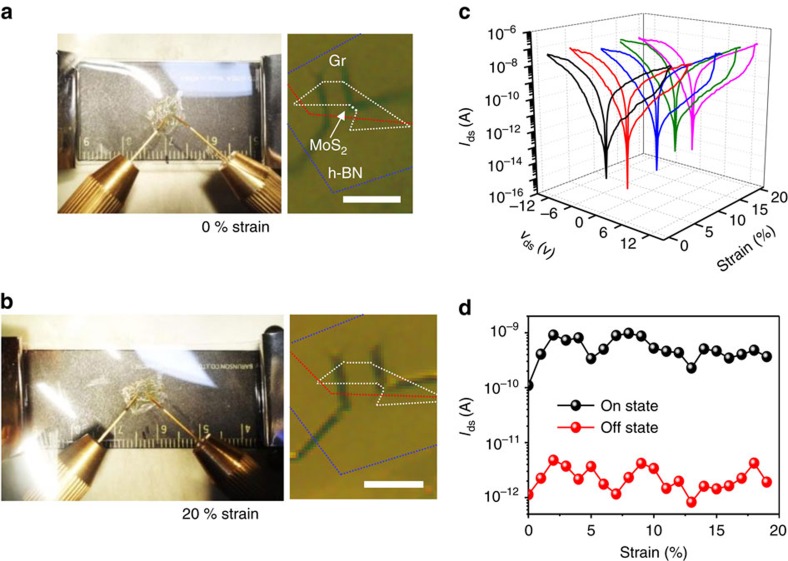
Stretchability of TRAM. (**a**–**b**) Optical images of TRAM on PI/PDMS stretchable substrate before and after stretching 20% along the length direction. Scale bars are 10 μm. The device is wrinkled or crumpled after transfer. The boundaries of MoS_2_, h-BN and graphene area are marked by white, blue and red colours, respectively. (**c**) *I*–*V* curve of the device on PI/PDMS before stretching (black curve) and after stretching at certain strains (coloured). (**d**) On-current (black) and off-current (red) with tensile strain at a given voltage of 1 V. Memory function disappears at 20% strain. A 10-nm thick h-BN layer was used for the tunnelling insulator in (**a**).

To further extract data for stretchability, we fabricated TRAM devices on a stretchable substrate of polyimide (PI)/polydimethylsiloxane (PDMS) (Fig. 7a,b). The formation of wrinkles of thin-layered materials^37^ was inevitable during fabrication process (see Methods section). Because of this, the on/off ratio was reduced to ∼10^3^ after transfer of the device onto PDMS substrate (Fig. 7c black curve). This reduction of on/off ratio is mainly attributed to mechanical degradation of van der Waals contacts or metal electrode caused during the transfer process (Supplementary Fig. 27a). Nevertheless, the *I*–*V* curve showed a stable memory hysteresis in the forward and reverse drain sweeps until 19% strain, and the on/off ratio was maintained at 10^3^ without degradation (Fig. 7d). The device fails to operate at a strain above 20%, where the leakage current of the tunnelling insulator surges (Supplementary Fig. 27b). It should be noted that the high stretchability of our device does not result from intrinsic properties of 2D materials but from the nature of the wrinkled structure^37^.

## Discussion

In summary, we have demonstrated a TRAM with two terminals, which is gate-free and blocking insulator-free device. In this way, our TRAM can achieve not only highly reliable memory performance, such as high on/off ratio up to 10^9^, long retention time (>10^4^ s), stable endurance (>10^5^ cycles) and multilevel feasibility, but also excellent stretchability with no appreciable electrical performance changes up to 19% strain because of the absence of a rigid blocking insulator and large apparent strain from the wrinkled structure on PI substrate. Our memory device can be useful for next-generation of wearable, body-attachable electronics in the near future.

## Methods

### The fabrication of MoS_2_/h-BN/Gr heterostructures

For the fabrication of the vertical heterostructures of the graphene/MoS_2_/h-BN device, the graphene was grown via chemical vapour deposition, transferred onto a Si/SiO_2_ (300 nm SiO_2_) substrate, and patterned into 10 × 50 μm strips as a floating gate using a photolithography and oxygen-plasma etching process. The h-BN and MoS_2_ flakes were then transferred onto the graphene strips through a dry transfer approach^22^. This method includes the mechanical exfoliation of the required flakes onto a dual-layer polymer stack polyvinyl alcohol (PVA) and polymethyl methacrylate. The bottom polymer (PVA) layer is dissolved in water and the resulting membrane is inverted and positioned above the target flake. The metal electrodes for probe contact were patterned on the MoS_2_ by e-beam deposition of Cr/Au (30/70 nm) followed by e-beam lithography.

For fabricating flexible devices on PET, we first stacked Gr/h-BN/MoS_2_ heterostructure on SiO_2_/Si wafer, and then floated them with polymethyl methacrylate binder on hydrofluoric acid etchant by etching SiO_2_ sacrifice layer. The stack was washed three times by floating in deionized water and then transferred onto PET substrate. The source and drain electrodes were then patterned by e-beam lithography.

For stretching test devices, a thin PI film was formed on the SiO_2_/Si wafer by spin-coating polyamic acid followed by annealing. The graphene/h-BN/MoS_2_ heterostructures and source–drain electrodes were formed by a dry transfer method and e-beam lithography, respectively, on the prepared PI film-coated Si wafer. A 10-nm thick h-BN layer was used for the tunnelling insulator. The TRAM-loaded PI film was detached from the Si wafer by etching the sacrificial SiO_2_ layer with hydrofluoric acid acid solution and then transferred onto the PDMS substrate.

### Characterization

A SPA400 (SEIKO) was used to record the AFM images. Electrical transport measurements were conducted with a probe station and source/measure units (Keithley 4200 and Agilent B2900A). For the stretching test, one side of the PI/PDMS substrate was fixed and the other side was pulled to stretch. All electrical measurements were performed in high vacuum (∼10^−6^ Torr).

### Electrostatic simulation

Device simulation was carried out by the Comsol Multiphysics package using the electrostatic module. The Laplace equation with given boundary conditions for voltages was numerically solved by the finite element method.

### Data availability

The data that support the findings of this study are available from the corresponding author upon request

## Additional information

**How to cite this article:** Vu, Q. A. *et al*. Two-terminal floating-gate memory with van der Waals heterostructures for ultrahigh on/off ratio. *Nat. Commun.* 7:12725 doi: 10.1038/ncomms12725 (2016).

## Supplementary Material

Supplementary Information Supplementary Figures 1-27, Supplementary Tables 1-2, Supplementary Notes 1-4, and Supplementary References

## References

[b1] Kahng D. & Sze S. M. A floating gate and its application to memory devices. Bell Syst. Tech. J. 1, 1288–1295 (1967).

[b2] Wong H.-S. P. & Salahuddin S. Memory leads the way to better computing. Nat. Nanotechnol. 10, 191–194 (2015). 25740127 10.1038/nnano.2015.29

[b3] Kent A. D. & Worledge D. C. A new spin on magnetic memories. Nat. Nanotechnol. 10, 187–191 (2015). 25740126 10.1038/nnano.2015.24

[b4] Sekitani T. . Organic nonvolatile memory transistors for flexible sensor arrays. Science 326, 1516–1519 (2009). 20007895 10.1126/science.1179963

[b5] Zhao C., Zhao C. Z., Taylor S. & Chalker P. R. Review on non-volatile memory with high-k dielectrics: Flash for generation beyond 32 nm. Materials 7, 5117–5145 (2014). 10.3390/ma7075117 PMC5455833 28788122

[b6] Ovshinsky S. R. Reversible electrical switching phenomena in disordered structures. Phys. Rev. Lett. 21, 1450–1453 (1968).

[b7] Lankhorst M. H. R., Ketelaars B. W. S. M. M. & Wolters R. A. M. Low-cost and nanoscale non-volatile memory concept for future silicon chips. Nat. Mater. 4, 347–352 (2005). 15765107 10.1038/nmat1350

[b8] Wuttig M. & Yamada N. Phase-change materials for rewriteable data storage. Nat. Mater. 6, 824–832 (2007). 17972937 10.1038/nmat2009

[b9] Strukov D. B., Snider G. S., Stewart D. R. & Williams R. S. The missing memristor found. Nature 453, 80–83 (2008). 18451858 10.1038/nature06932

[b10] Yang J. J., Strukov D. B. & Stewart D. R. Memristive devices for computing. Nat. Nanotechnol. 8, 13–24 (2013). 23269430 10.1038/nnano.2012.240

[b11] Hartmanet T. E. Electrical conduction through SiO films. J. Appl. Phys. 37, 2468 (1966).

[b12] Wong H. S. P. . Phase change memory. Proc. IEEE 98, 2201–2227 (2010).

[b13] Yeh C.-C. . Frenkel–Poole trap energy extraction of atomic layer deposited Al_2_O_3_ and Hf_*x*_Al_*y*_O thin films. Appl. Phys. Lett. 91, 113521 (2007).

[b14] Ielmini D. Threshold switching mechanism by high-field energy gain in the hopping transport of chalcogenide glasses. Phys. Rev. B 78, 035308 (2008).

[b15] Cheng C. H., Chen P. C., Wu Y. H., Yeh F. S. & Chin A. Long-endurance nanocrystal TiO_2_ resistive memory using a TaON buffer layer. IEEE Electron Device Lett. 32, 1749–1751 (2011).

[b16] Geim A. K. & Grigorieva I. V. Van der Waals heterostructures. Nature 499, 419–425 (2013). 23887427 10.1038/nature12385

[b17] Britnell L. . Field-effect tunneling transistor based on vertical graphene heterostructures. Science 335, 947–950 (2012). 22300848 10.1126/science.1218461

[b18] Yu W. J. . Vertically stacked multi-heterostructures of layered materials for logic transistors and complementary inverters. Nat. Mater. 12, 246–252 (2013). 23241535 10.1038/nmat3518 PMC4249642

[b19] Georgiou T. . Vertical field-effect transistor based on graphene–WS_2_ heterostructures for flexible and transparent electronics. Nat. Nanotechnol. 8, 100–103 (2013). 23263726 10.1038/nnano.2012.224

[b20] Cui X. . Multi-terminal transport measurements of MoS_2_ using a van der Waals heterostructure device platform. Nat. Nanotechnol. 10, 534–540 (2015). 25915194 10.1038/nnano.2015.70

[b21] Britnell L. . Strong light–matter interactions in heterostructures of automatically thin films. Science 340, 1311–1314 (2013). 23641062 10.1126/science.1235547

[b22] Yu W. J. . Highly efficient gate-tunable photocurrent generation in vertical heterostructures of layered materials. Nat. Nanotechnol. 8, 952–958 (2013). 24162001 10.1038/nnano.2013.219 PMC4249654

[b23] Withers F. . Light-emitting diodes by band-structure engineering in van der Waals heterostructures. Nat. Mater. 14, 301–306 (2015). 25643033 10.1038/nmat4205

[b24] Withers F. . WSe_2_ light-emitting tunneling transistors with enhanced brightness at room temperature. Nano Lett. 15, 8223–8228 (2015). 26555037 10.1021/acs.nanolett.5b03740

[b25] Lee C.-H. . Atomically thin p–n junctions with van der Waals heterointerfaces. Nat. Nanotechnol. 9, 1–29 (2014). 25108809 10.1038/nnano.2014.150

[b26] Akinwande D., Petrone N. & Hone J. Two-dimensional flexible nanoelectronics. Nat. Commun. 5, 5678 (2014). 25517105 10.1038/ncomms6678

[b27] Das S., Gulotty R., Sumant A. V. & Roelofs A. All two-dimensional, flexible, transparent, and thinnest thin film transistor. Nano Lett. 14, 2861–2866 (2014). 24754722 10.1021/nl5009037

[b28] Dean C. R. . Boron nitride substrates for high-quality graphene electronics. Nat. Nanotechnol. 5, 722–726 (2010). 20729834 10.1038/nnano.2010.172

[b29] Choi M. S. . Controlled charge trapping by molybdenum disulphide and graphene in ultrathin heterostructured memory devices. Nat. Commun. 4, 1624 (2013). 23535645 10.1038/ncomms2652

[b30] Simone B., Daria K. & Andras K. Nonvolatile memory cell based on MoS_2_/graphene heterostructures. ACS Nano 7, 3246–3252 (2013). 23510133 10.1021/nn3059136

[b31] Lee G. H. . Electron tunneling through atomically flat and ultrathin hexagonal boron nitride. Appl. Phys. Lett. 99, 243114 (2011).

[b32] Bessonov A. . Layered memristive and memcapacitive switches for printable electronics. Nat. Mater. 14, 199–204 (2014). 25384168 10.1038/nmat4135

[b33] Yubao L. . Electronic two-terminal bistable graphitic memories. Nat. Mater. 7, 966–971 (2008). 19011617 10.1038/nmat2331

[b34] Lee C. . Measurement of the elastic properties and intrinsic strength of monolayer graphene. Science 321, 385–388 (2008). 18635798 10.1126/science.1157996

[b35] Bertolazzi S., Brivio J. & Kis A. Stretching and breaking of ultrathin MoS_2_. ACS Nano 5, 9703–9709 (2011). 22087740 10.1021/nn203879f

[b36] Andrew R., Mapasha R., Ukpong A. & Chetty N. Mechanical properties of graphene and boronitrene. Phys. Rev. B 85, 1–9 (2012).

[b37] Chae S. H. . Transferred wrinkled Al_2_O_3_ for highly stretchable and transparent graphene–carbon nanotube transistors. Nat. Mater. 12, 403–409 (2013). 23455851 10.1038/nmat3572

