# Supplementary material for: Two-terminal floating-gate memory with van der Waals heterostructures for ultrahigh on/off ratio
Source: Nat Commun. 2016 Sep 2;7:12725. doi: 10.1038/ncomms12725 (PMC5025799; doi:10.1038/ncomms12725)
Supplement: Supplementary Information — Supplementary Figures 1-27, Supplementary Tables 1-2, Supplementary Notes 1-4, and Supplementary References [file ncomms12725-s1.pdf]

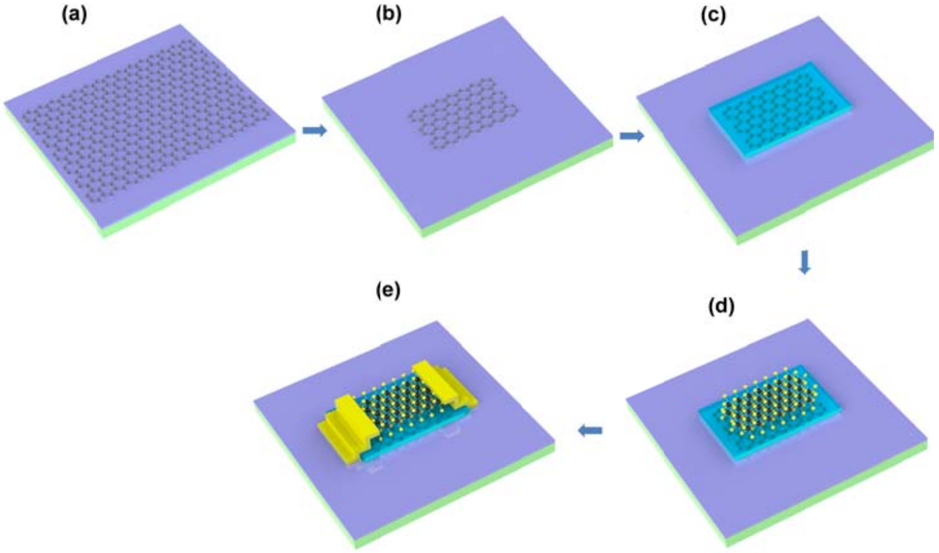

Supplementary Figure 1 | Schematic illustration of fabrication procedure of MoS_2/h-BN/graphene heterostructures. a, CVD-grown monolayer graphene was transferred on a 300-nm SiO_2-covered silicon substrate. **b,** Graphene was patterned into graphene strips by oxygen plasma etching using photoresist as an etching mask. **c–d**, h-BN and MoS_2 were transferred onto the graphene through a dry transfer technique. **e,** Cr/Au (30 nm/70 nm) electrodes were patterned using e-beam lithography.

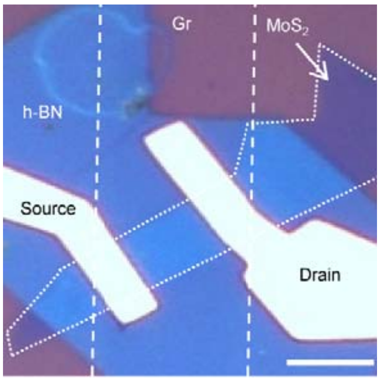

Supplementary Figure 2 | Optical image of the TRAM, the scale bar is 5 μm.

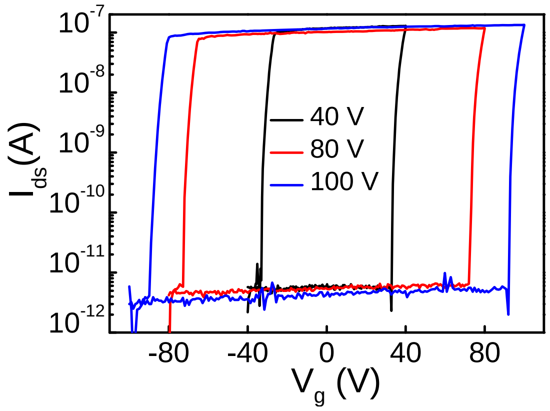

Supplementary Figure 3 | Gate dependence of the on and off state of device with an h-BN thickness of 7.5 nm at V_{ds} = 0.01 V.

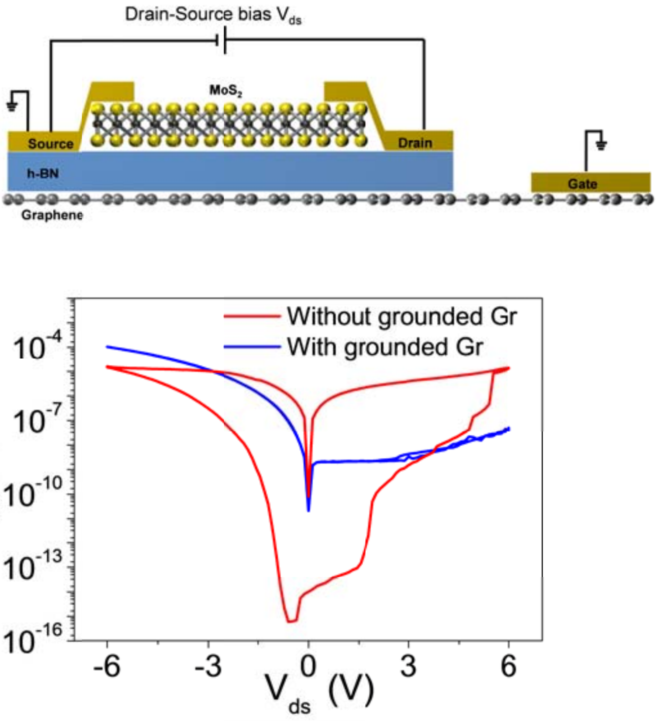

Supplementary Figure 4 | I-V characteristic of TRAM with and without grounded graphene. The hysteresis of our devices originates from charging in graphene and the real current flows through MoS_2 channel. In order to demonstrate this, graphene is grounded. Now the IV sweep from -6 V to +6 V includes both current flow through MoS_2 channel and tunneling through BN. Because of the work function difference between Au electrode and graphene, tunneling current is dominant at high voltage and channel effect is dominant at low voltage. More importantly, no hysteresis is observed during this sweep. This implies no additional charge accumulations near the electrode-MoS_2 contact and therefore no influence to the memory characteristics we demonstrated with floating graphene.

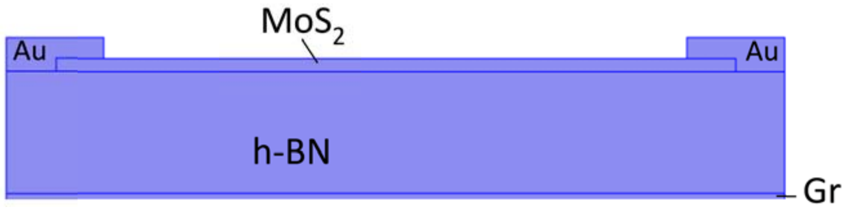

Supplementary Figure 5 | Geometry of the simulation model

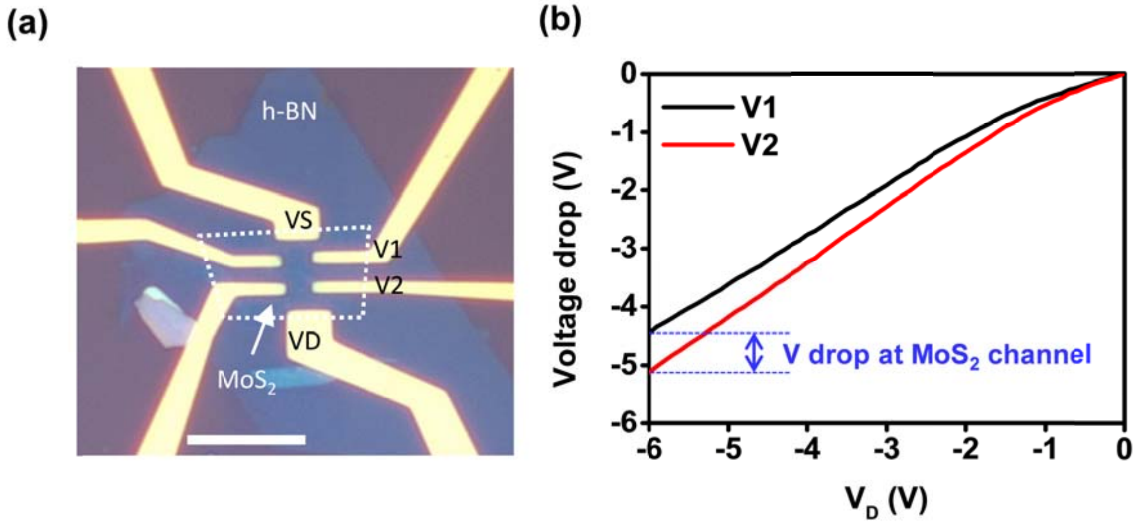

Supplementary Figure 6 | a, Optical image of four probes MoS_2 channel. **b**, Measured voltage drop at MoS_2 channel. The scale bar is 10 μm.

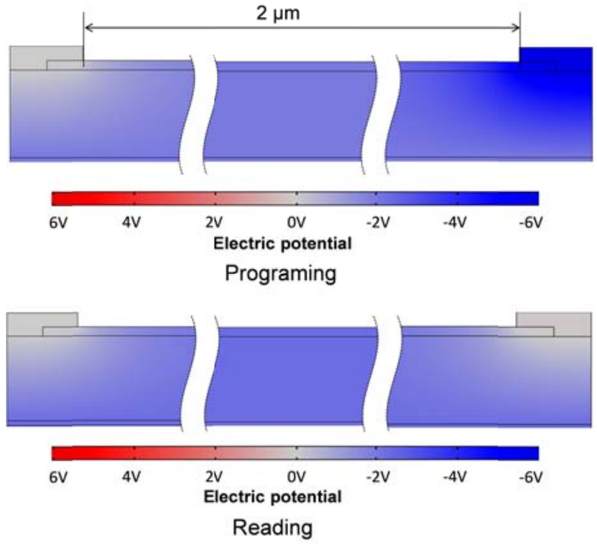

Supplementary Figure 7 | Electrostatic potential simulation at Programing state (top) and Reading in Programing state (bottom) for channel length of 2 μm.

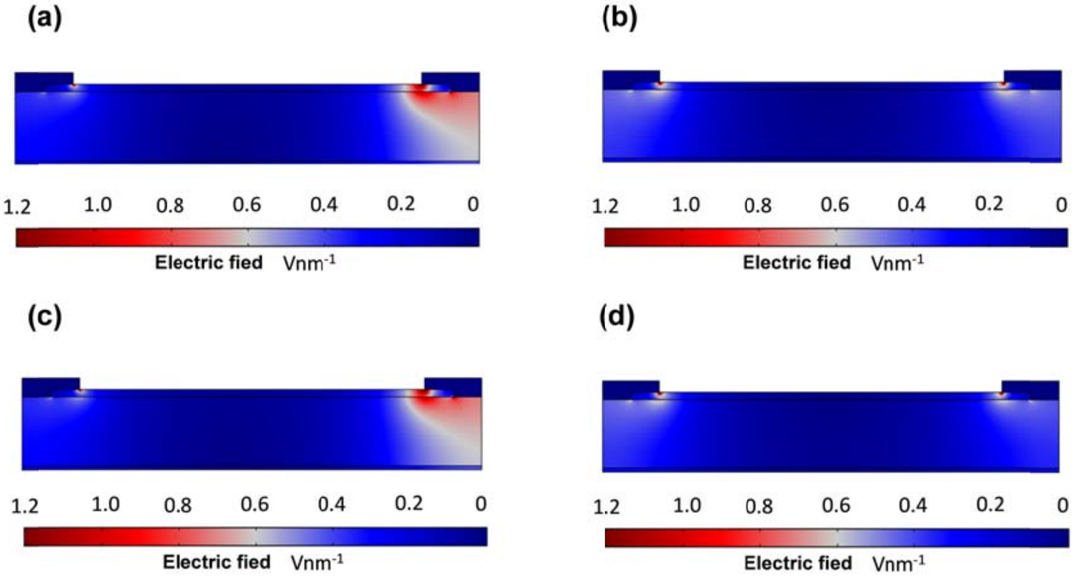

Supplementary Figure 8 | Electrical distribution. Electric field distribution in the device in (**a**) program, (**b**) read in program state, (**c**) erase, and (**d**) read in erase state.

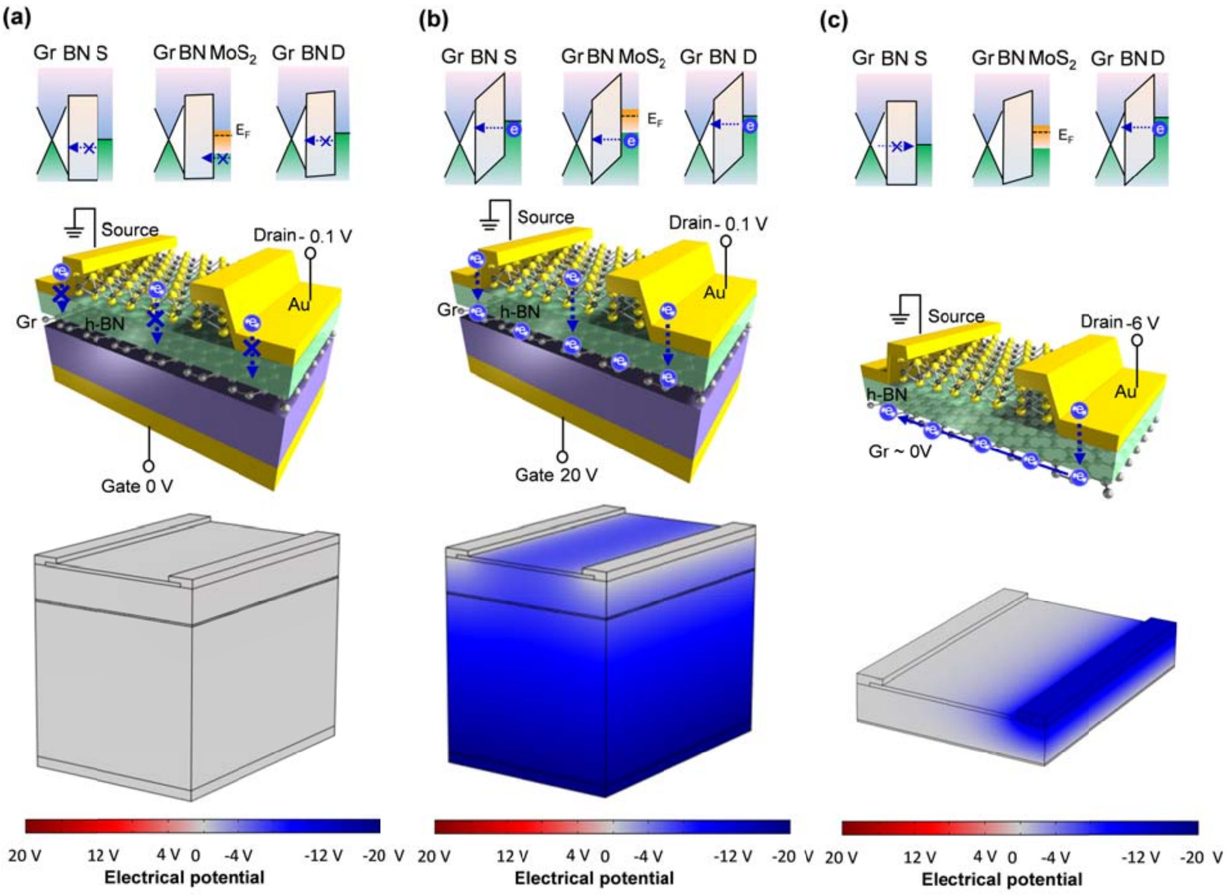

Supplementary Figure 9 | Numerical simulation of potential distribution in Flash, TRAM and the schematic band diagram. Schematic illustration of band diagram (top), memory operation (middle), and simulated electrostatic potential (bottom) at the states of a, without applying gate bias in Flash (V_g = 0 V, V_{ds} = -0.1 V), b, applying gate bias in Flash (V_g = 20 V, V_{ds} = -0.1 V), c, biasing in TRAM (V_{ds} = -6 V), The dashed line arrows indicate the tunneling direction of electrons from drain into graphene, the solid line arrows indicate the spreading of electrons at the graphene after tunneling.

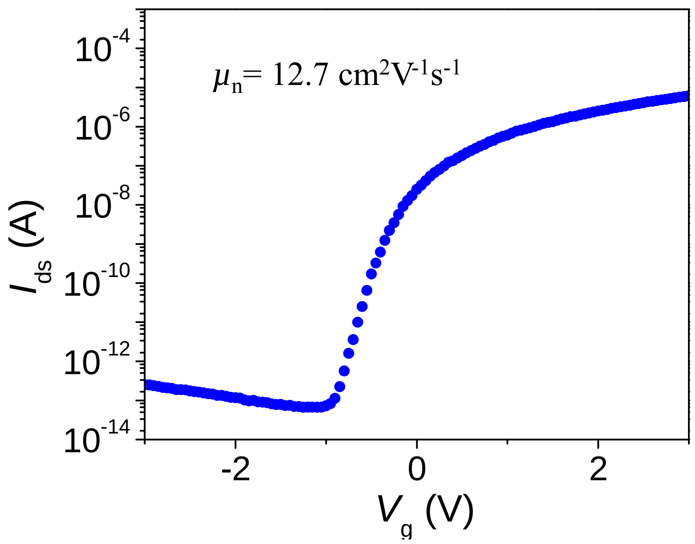

Supplementary Figure 10 | I-V curve and mobility of the MoS_2 transistor with h-BN dielectric layer and graphene gate electrode.

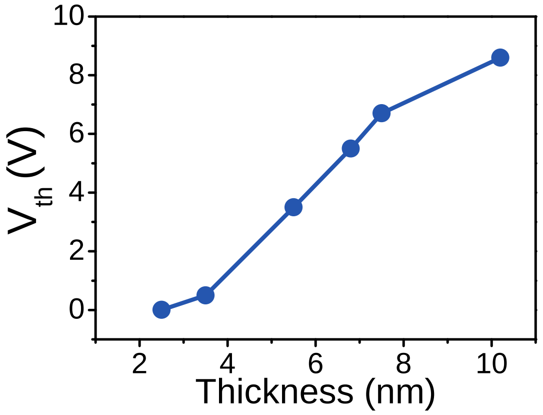

Supplementary Figure 11 | Change of threshold voltage for various thicknesses of h-BN layer. Threshold voltages were taken at 10^{-13} A of the tunneling current measured between the drain electrode and graphene.

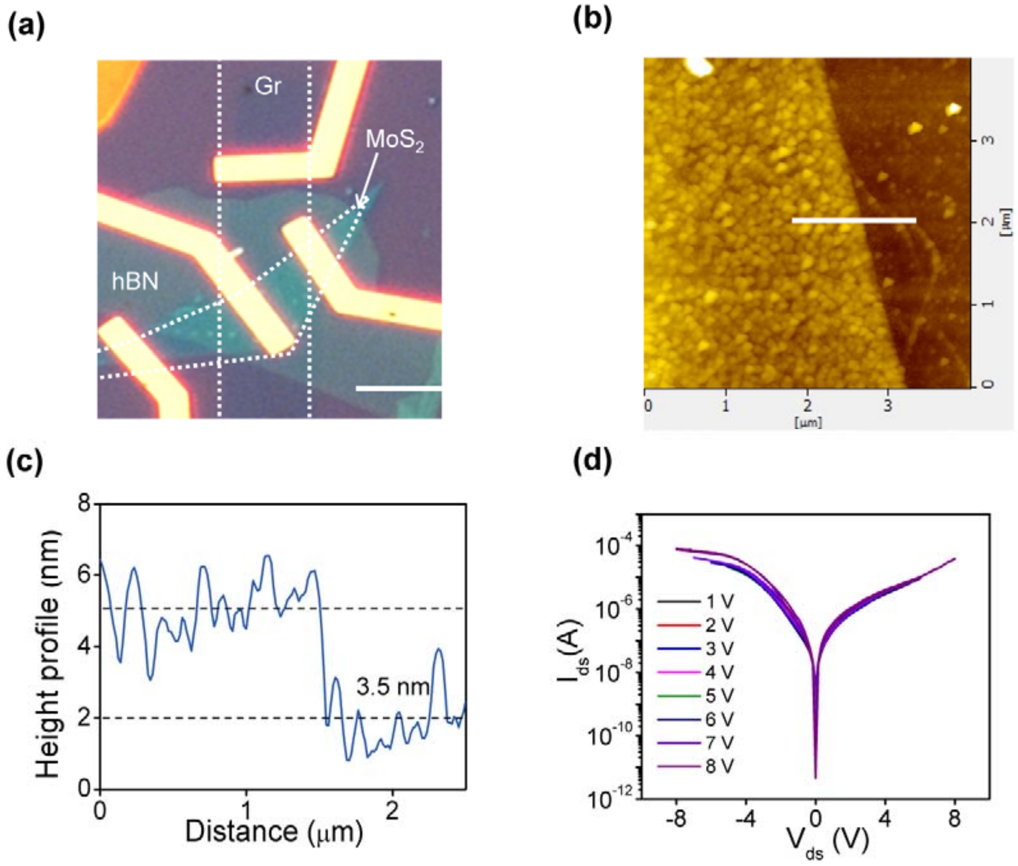

Supplementary Figure 12 | Characteristics of the device with an h-BN thickness of 3.5 nm. **a,** Optical image of the device with a 3.5-nm thick h-BN; the scale bar is 10 μm. **b,** AFM image of h-BN. **c,** Height profile of an h-BN flake extracted from AFM measurement. **d,** I-V curves at different V_{ds} sweep ranges.

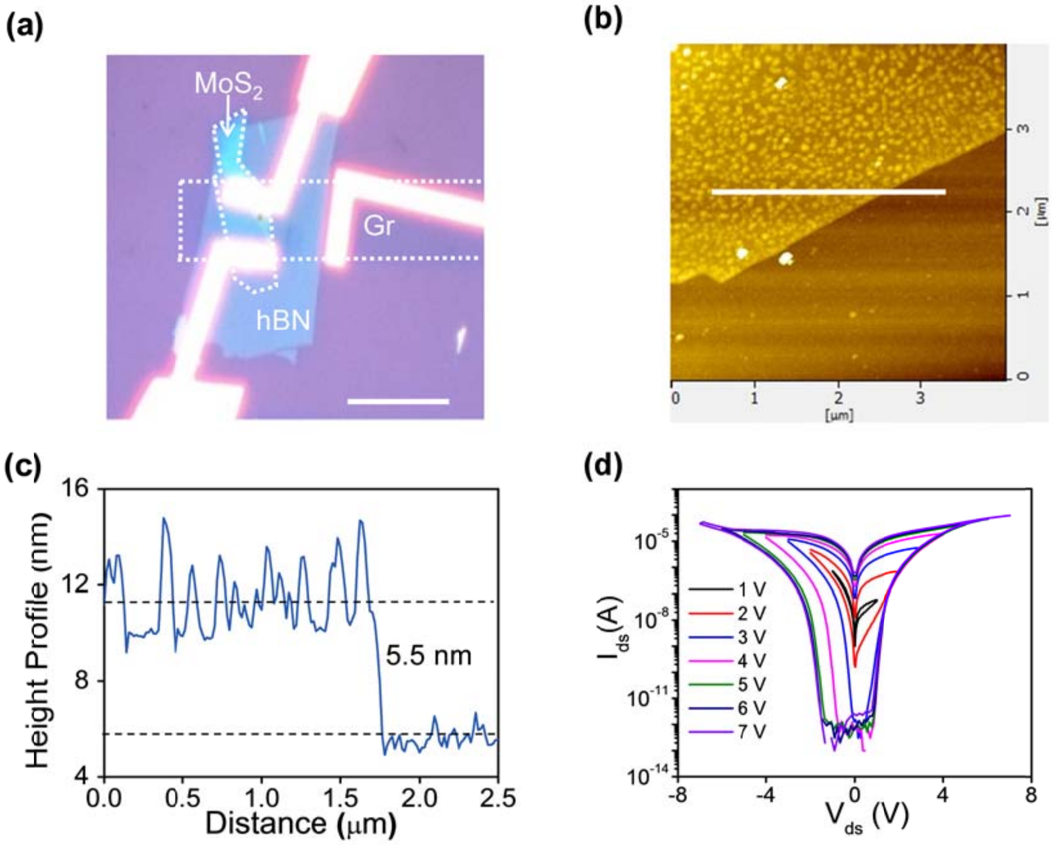

Supplementary Figure 13 | Characteristics of the device with an h-BN thickness of 5.5 nm. **a,** Optical image of the device with a 5.5-nm thick h-BN; the scale bar is 10 μm. **b,** AFM image of h-BN. **c,** Height profile of an h-BN flake extracted from AFM measurement. **d,** I-V curves at different V_{ds} sweep ranges.

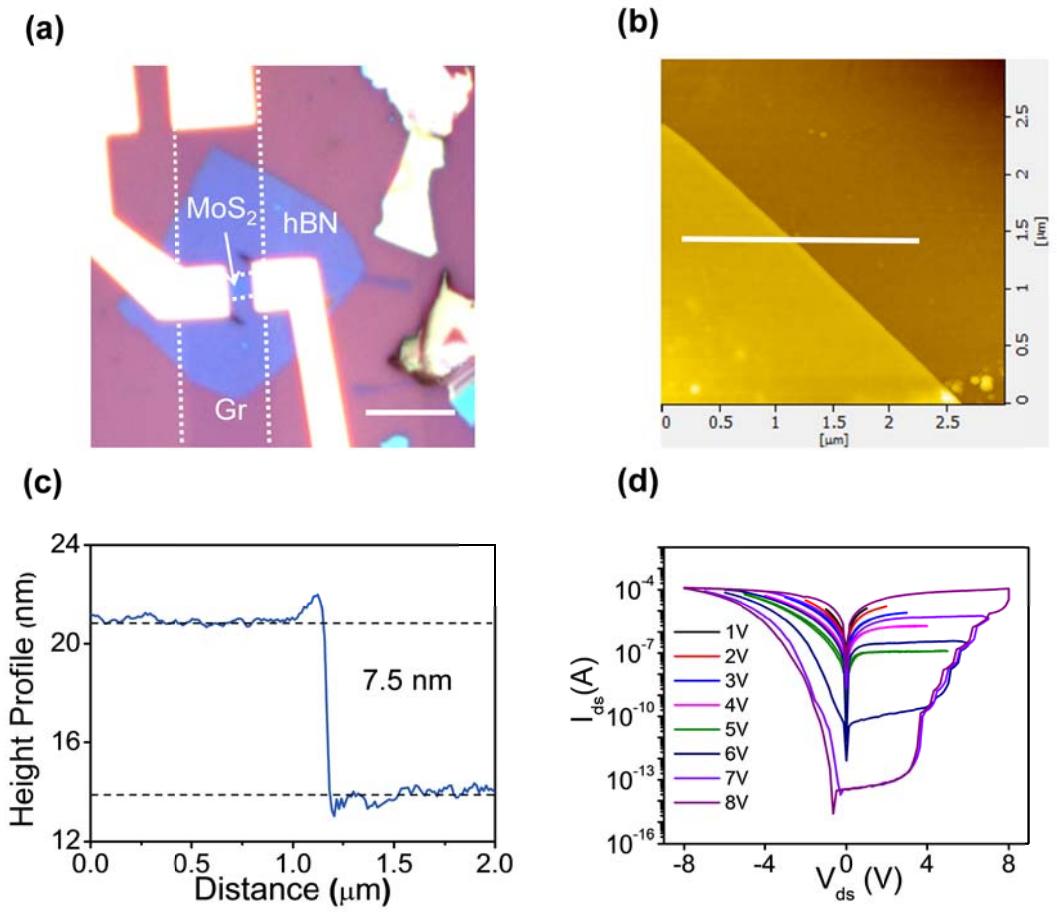

Supplementary Figure 14 | Characteristics of the device with an h-BN thickness of 7.5 nm. **a,** Optical image of the device with a 7.5-nm thick h-BN; the scale bar is 10 μm. **b,** AFM image of h-BN. **c,** Height profile of an h-BN flake extracted from AFM measurement. **d,** I-V curves at different V_{ds} sweep ranges.

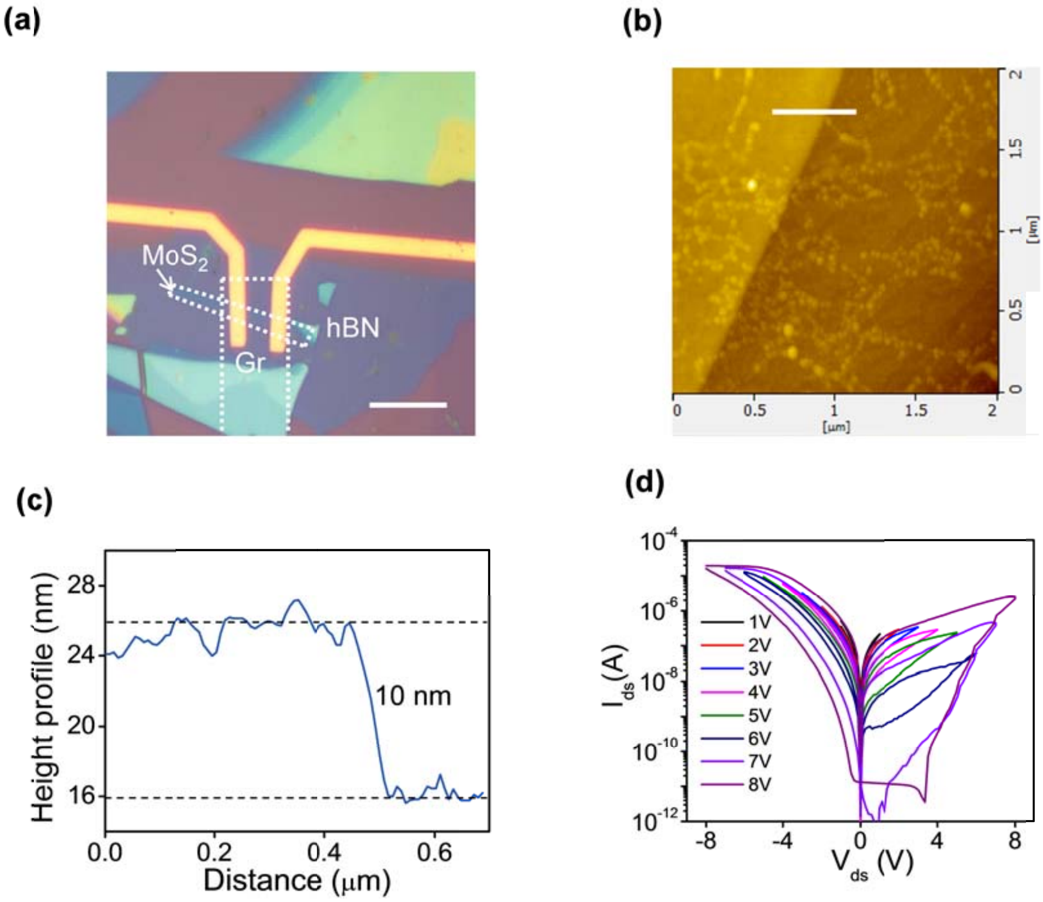

Supplementary Figure 15 | Characteristics of the device with an h-BN thickness of 10 nm. a, Optical image of the device with a 10-nm thick h-BN; the scale bar is 10 μm. **b,** AFM image of h-BN. **c,** Height profile of an h-BN flake extracted from AFM measurement. **d,** I-V curves at different V_{ds} sweep ranges. A small hysteresis is shown at 6 V; the hysteresis is larger with increasing bias voltage.

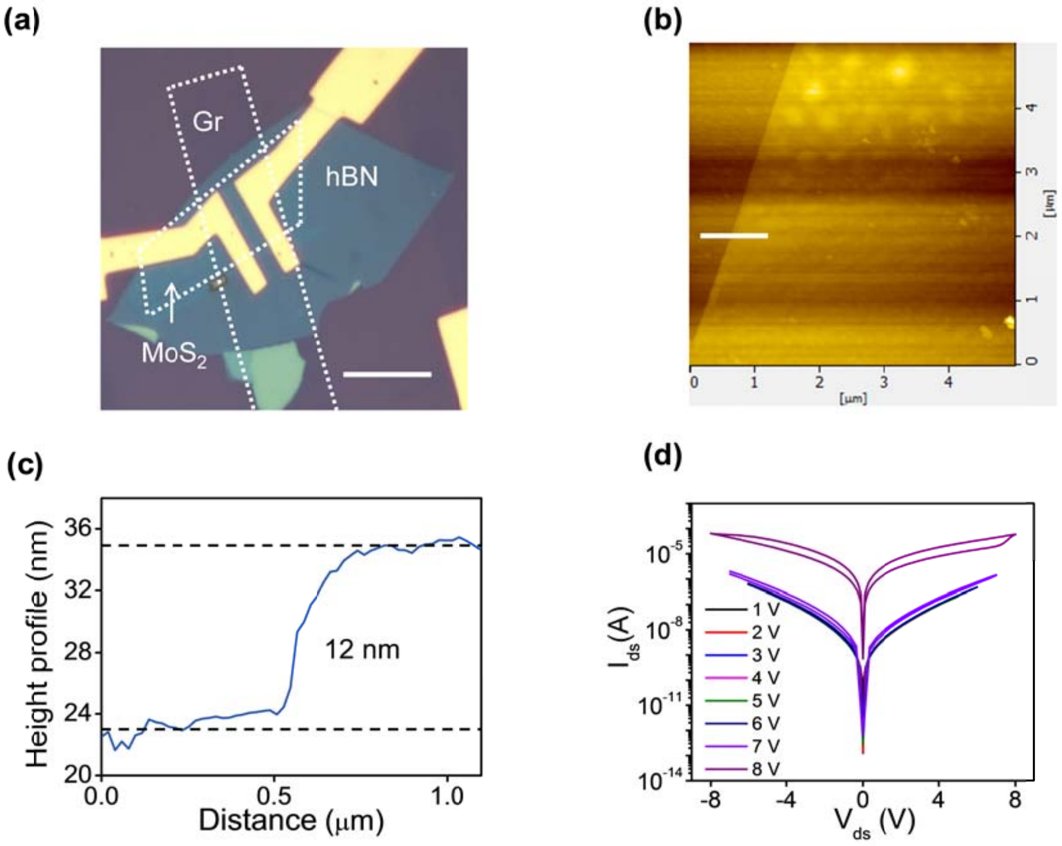

Supplementary Figure 16 | Characteristics of the device with an h-BN thickness of 12 nm. **a,** Optical image of the device with a 12-nm thick h-BN; the scale bar is 10 μm. **b,** AFM image of h-BN. **c,** Height profile of an h-BN flake extracted from AFM measurement. **d,** I-V curves at different V_{ds} sweep ranges.

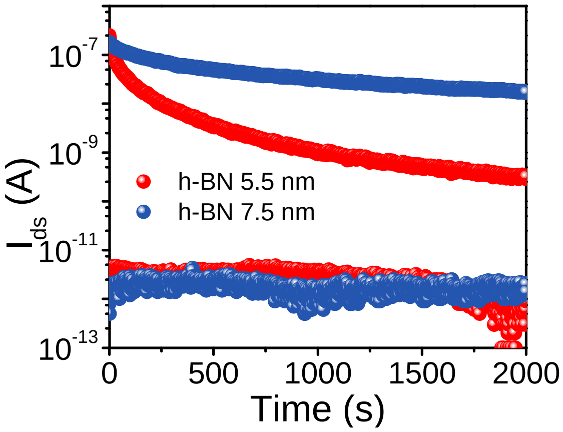

Supplementary Figure 17 | Retention time of TRAM with h-BN thicknesses of 5.5 nm and 7.5 nm. Retention time of TRAM with h-BN thicknesses of 5.5 nm (red dots) and 7.5 nm (blue dots) for 2000 s, pulse height of $V_{ds} = \pm 8$ V for 5 s, $V_{reading} = 0.01$ V.

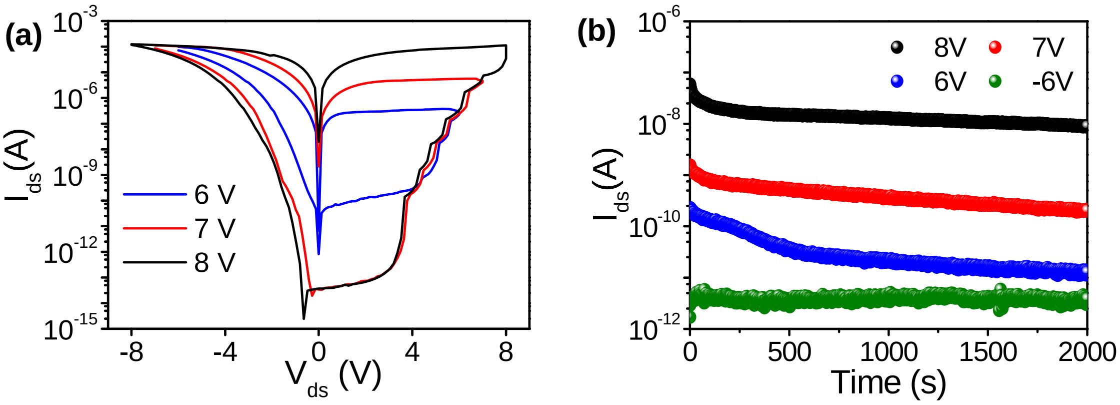

Supplementary Figure 18 | Multi level feasibility. a, I-V characteristics of the same device at different V_{ds} sweep ranges of ±6 V, ±7 V, and ±8 V to demonstrate multi-level memory performance. **b,** Retention characteristics at different erase pulse voltages of 6 V (blue), 7 V (red), and 8 V (black) and program pulse voltages of −6 V (green), $V_{reading} = 0.01$ V.

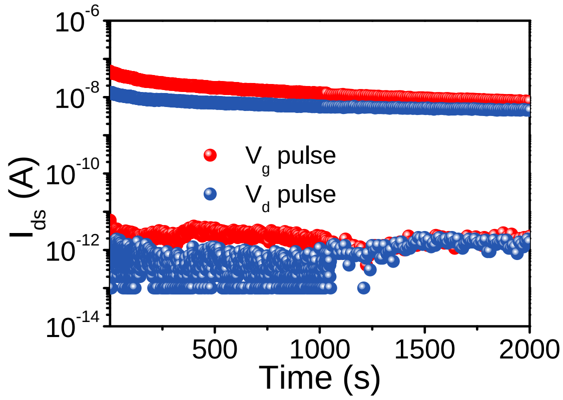

Supplementary Figure 19 | Comparison of the charge retention between gate pulse and drain pulse. Pulse heights of gate and drain are ±15 V and ±8 V, respectively, at a pulse width of 5 s.

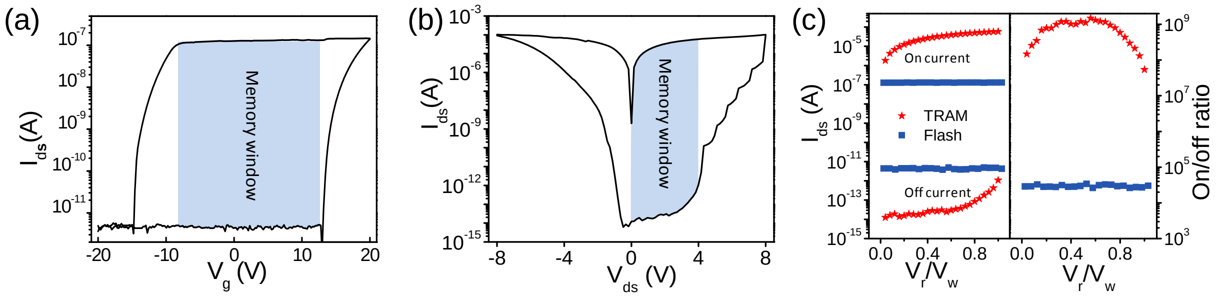

Supplementary Figure 20 | Comparison Off-current, on/off ratio between TRAM and Flash **a,** I-V characteristics of flash memory, **b,** TRAM. **c,** On, off current (left panel) and on/off ratio of TRAM and Flash memory (right panel).

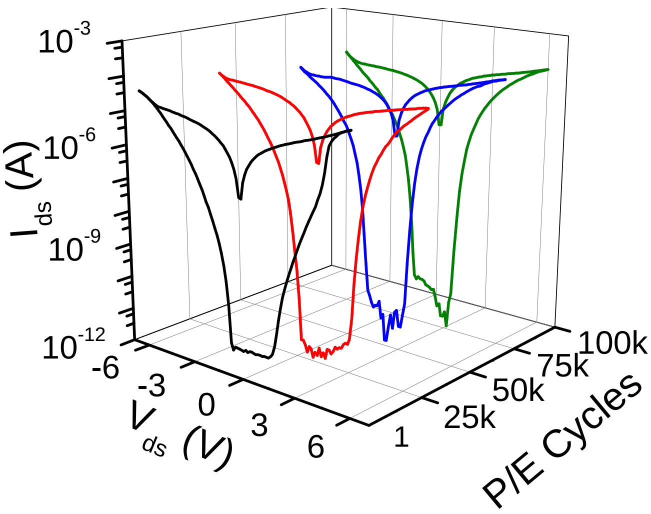

Supplementary Figure 21 | Endurance characteristics of the TRAM. Program and erase were carried out by −6 V and +6 V with a pulse width of 0.1 s and a reading voltage of 0.1 V.

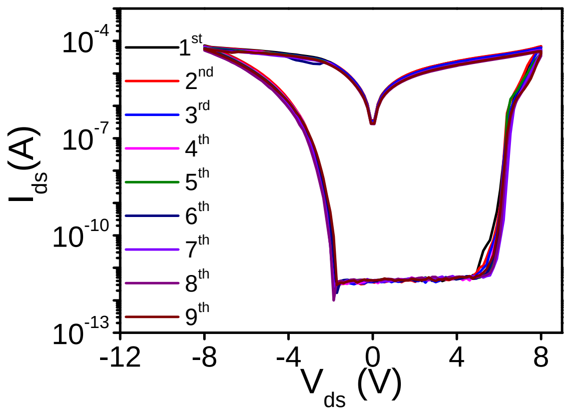

Supplementary Figure 22 | Reproducibility. I-V curves of multiple sweeps (nine sweeps) of the TRAM.

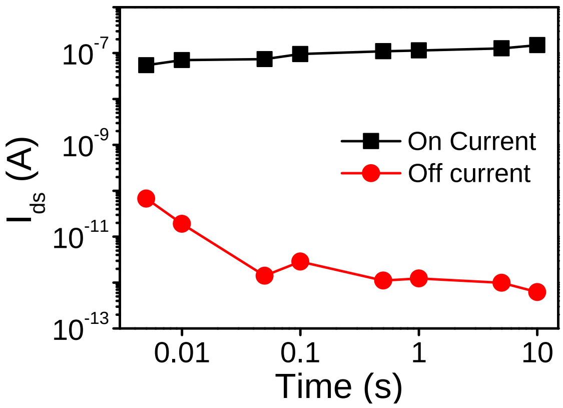

Supplementary Figure 23 | Speed of the device.

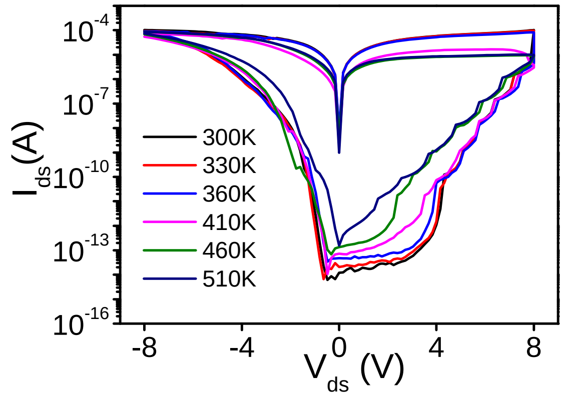

Supplementary Figure 24 |Temperature stability.

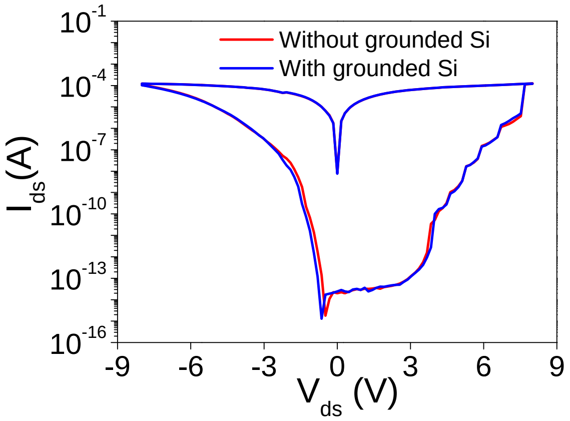

Supplementary Figure 25 | I-V characteristic of TRAM with and without grounded graphene. Both TRAMs of grounded and no grounded Si were shown same memory behavior. Moreover, the memory behavior of our TRAM is also shown on PET and PI polymer insulating substrate (Figure 5), confirming that our TRAM can operate without a control gate.

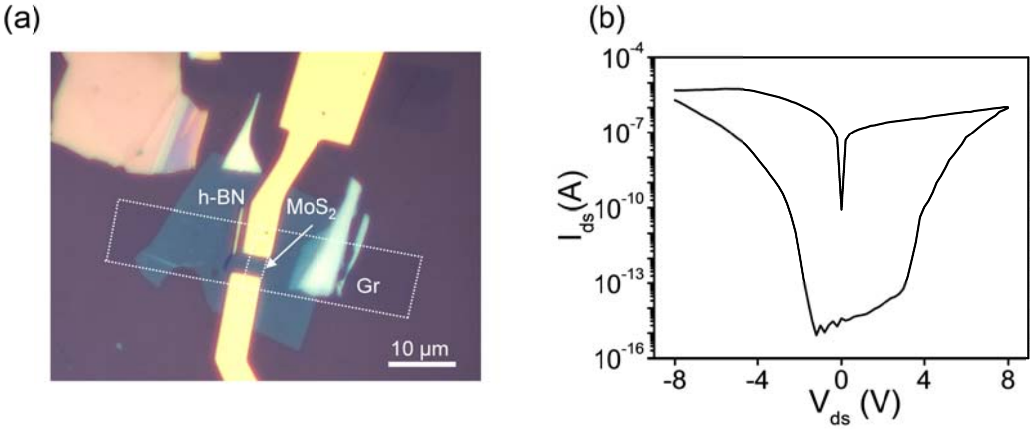

Supplementary Figure 26 | Influence of metal pad contact to the graphene layer and capacitive coupling between the metal electrodes/pads and the Si substrate on the performance of the device. a, Optical image and **b,** I-V characteristic of memory device without a metal contact to the graphene floating gate for h-BN thickness of 8 nm. Channel length and channel width of the device are 5 and 2 μm, respectively. There was no difference in the I-V characteristics between with and without metal pad.

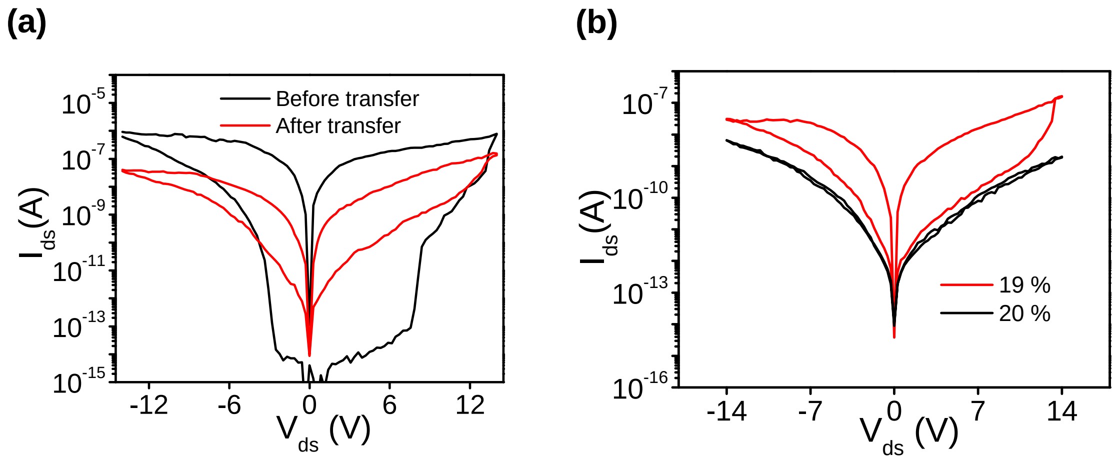

Supplementary Figure 27 | The degradation of the performance after transfer and the device failed at high strain in the stretchability test. a, I-V curve of the TRAM with 10 nm thick h-BN on PI/ SiO_2/Si substrate before transfer and after transfer onto PDMS substrate. The mechanical degradation of electrode may cause the reduction of on/off ratio. **b,** The device failed to operate at 20% strain.

Supplementary Table 1 | Parameters of the geometries and dielectric constants

Material/parameter	Thickness	Width	Dielectric constant
**Au**	1 nm	5 nm	6.9[(2)]
**MoS_2**	0.65 nm	30 nm	7.3[(3)]
**h-BN**	5.5 nm	40 nm	5.0[(4)]
**Gr**	0.34 nm	40 nm	5.0[(5,6)]

Supplementary Table 2 | Voltages applied for each sweep used in the simulation

Voltage/State	Program	Read in Program state	Erase	Read in Erase state
**Drain**	−6 V	0.1 V	6 V	0.1 V
**Gr**	-2.2 V	−2.27 V	2.2 V	2.33 V
**Source**	grounded	grounded	grounded	grounded

Supplementary Note 1 | Electrostatic potential simulation at different states

Geometry and material parameters of the simulation model are given as Supplementary Figure 5, Supplementary Tables 1 and Supplementary Table 2. Typical channel length of our device is about 2 μm. In fact, we performed two simulations for two channel lengths: 30 nm and 2 μm. The potential distribution in both channel lengths is almost similar (Figure 2 and Supplementary Figure S6). Potential distribution of 2 μm channel cannot illustrate in single image due to large difference between channel length and thickness (2000/7 nm). Therefore, we used simulation model with the channel length of 30 nm for better eye capturing. In this small difference between channel length and thickness (30/7 nm), electrostatic potential distribution can illustrate in single image.

The floating gate potential V_{FG} estimated by Cappelletti[1] is:

$$V_{\mathrm{FG}} = \frac{Q_{\mathrm{FG}}}{C_{\mathrm{T}}} + \frac{C_{\mathrm{C}}V_{\mathrm{C}}}{C_{\mathrm{T}}} + \frac{C_{\mathrm{S}}V_{\mathrm{S}}}{C_{\mathrm{T}}} + \frac{C_{\mathrm{D}}V_{\mathrm{D}}}{C_{\mathrm{T}}} + \frac{C_{\mathrm{B}}V_{\mathrm{B}}}{C_{\mathrm{T}}} \qquad \text{(Equation 1)}$$

where Q_{FG} is the charge stored in the floating gate, C_C, C_S, C_D, and C_B are the capacitances between FG and control gate, source, drain and semiconductor channel, respectively, and C_T is the total capacitance ($C_{\mathrm{T}} = C_{\mathrm{C}} + C_{\mathrm{S}} + C_{\mathrm{D}} + C_{\mathrm{B}}$). V_C, V_S, V_D, and V_B are the potentials of control gate, drain, source, and semiconductor channel, respectively.

Our device has no control gate, and the source is connected to the ground, which leads to zero for V_C and V_S. Therefore, the Supplementary Equation 1 becomes:

$$V_{\mathrm{FG}} = \frac{Q_{\mathrm{FG}}}{C_{\mathrm{T}}} + \frac{C_{\mathrm{D}}V_{\mathrm{D}}}{C_{\mathrm{T}}} + \frac{C_{\mathrm{B}}V_{\mathrm{B}}}{C_{\mathrm{T}}} \qquad \text{(Equation 2)}$$

The capacitance is defined to:

$$C = \varepsilon_{\mathrm{r}} C_0 = \frac{\varepsilon_{\mathrm{r}}\varepsilon_0 A}{d} \qquad \text{(Equation 3)}$$

where ε_r is relative permittivity, ε_0 is absolute permittivity, A is capacitor area, and d is thickness of insulator. All parameters of ε_r, ε_0, and d are the same in C_S, C_D, and C_B. Capacitance of the drain electrode C_D is in general very small compared to channel capacitance due to small contact area and therefore, C_D/C_T is small and the potential of the floating gate is nearly zero if no charge is accumulated in the floating gate. In our device, the area of contact electrode is not negligible

due to relatively large pattern area of the drain contact. We have measured overlapped areas between graphene - metal electrodes (drain or source) and graphene – MoS_2 from the optical images (Supplementary Figures 2, 14) which were same areas, it leads to:

$$C_D \cong C_s \cong C_B \cong \frac{1}{3}C_T \qquad \text{(Equation 4)}$$

By inserting equation (4) into equation (2), V_{FG} can be obtained as:

$$V_{FG} = \frac{Q_{FG}}{C_T} + \frac{1}{3}V_D + \frac{1}{3}V_B \qquad \text{(Equation 5)}$$

At the programing state (Fig. 2a) of V_D = -6 V, the charge in floating gate is zero (Q_{FG} = 0), the voltage drop in MoS_2 channel was measured to -0.6 V (0.2 V/μm) (Supplementary Figure 6), which results in V_{FG} = -2.2 V. At the reading state of program (Fig. 2b) with V_D = 0.1 V, the carrier density in the floating gate (Q_{FG}) is $-1.22 \times 10^{13}\text{cm}^{-2}$ (Supplementary Note 2), leading the V_{FG} = -2.27 V. By this way, we obtained the floating gate potential at the erasing state (V_D =6 V) and reading sate of erase (V_D =0.1 V, Q_{FG} = $1.22 \times 10^{13}\text{cm}^{-2}$) to 2.2 V and 2.33 V, respectively (Supplementary Table 2).

Electrostatic potential and electric field simulation were performed with those V_{FG} (Figure 2 and Supplementary Figure 8). Asymmetric potential of 2.2 V at FG/source and 3.8 V at FG/drain were formed at the programing and erasing states, which allows asymmetric charge tunneling at the drain electrode to FG.

Supplementary Note 2 | Fundamental difference between Flash and TRAM

To understand the fundamental difference in mechanism of our TRAM and Flash, we performed electrostatic simulation of Flash memory by adding a SiO_2 blocking oxide thickness of 30 nm and Au control gate of 5 nm. In Flash, without gate pulse, charges (electrons and holes) are not able to tunnel into graphene floating layer, small voltage applied between source and drain (-0.1 V) only drives current in MoS_2 channel as demonstrated by schematics in Fig. S8a (top and middle panels), there is no electrical potential between source, drain electrodes and floating gate (Supplementary Figure 8a bottom panel). When large bias (20 V in our experiment and simulation) were applied at control gate, large electric potential drop between MoS_2 channel, source, drain electrode and floating gate, leads to charges start tunneling from channel and source, drain electrode (Supplementary Figure 8b).

Meanwhile, our TRAM's mechanism is totally different due to absence of blocking insulator and back gate electrode. When we apply the source-drain bias (-6 V and 6 V), large electric potential is established between drain and floating gate. Charges only tunnel from drain (not from channel or source) into graphene floating gate (as demonstrated in Supplementary Figure 8c) and they are blocked to tunnel out to source. The operation principle of our TRAM is well distinguished with Flash.

Supplementary Note 3 | Carrier concentration calculation

We calculate the carrier concentration of MoS_2 in on state based on the on current level in Fig. 1e

Drift current density can be calculated by equation:

$$J = \mathrm{q}n\mu_{\mathrm{n}}E + \mathrm{q}n\mu_{\mathrm{p}}E \qquad \text{(Equation 6)}$$

Where, J is the current density, q is charge of the electron, E is the electric field between source and drain, μn and μp are the mobility of electron and hole, respectively.

MoS_2 is n-type material, main carriers are electrons. For simple calculation, drift current density generated by holes can be ignored, the Supplementary Equation 6 becomes:

$$J = \mathrm{q}n\mu_{\mathrm{n}}E \qquad \text{(Equation 7)}$$

Carrier concentration can be calculated by:

$$n = \frac{J}{\mathrm{q}\mu_{\mathrm{n}}E} = \frac{Il}{w\mathrm{q}\mu_{\mathrm{n}}V_{\mathrm{ds}}} \qquad \text{(Equation 8)}$$

Where, I is the current, l, w are the channel length and channel width, respectively.

To calculate the mobility, we use the equation:

$$\mu_{\mathrm{n}} = \frac{lG_m}{wC_iV_{\mathrm{ds}}} \qquad \text{(Equation 9)}$$

Where, G_m and C_i are transconductance and capacitance per unit area, respectively.

Based on the I-V characteristic of our device (Supplementary Figure 10), electron mobility in MoS_2 field effect transistor in is calculated around 12.7 $cm^2V^{-1}s^{-1}$. The current in on state in Fig. 1e is 1.4×10^{-6} A under a source-drain bias V_{ds} of 0.1 V. The channel length and channel width of MoS_2 are 3 μm and 2 μm, respectively. Carrier concentration in MoS_2 is calculated:

$$n_{\mathrm{MoS_2}} = \frac{1.4\times 10^{-6}\times 3}{2\times 1.6\times 10^{-19}\times 12.7\times 0.1} = 1.02\times 10^{17}(\mathrm{m^{-2}}) = 1.02\times 10^{13}(\mathrm{cm^{-2}})$$

Carrier concentration in graphene can be calculated by equation:

$$n_{\mathrm{Gr}} = n_{\mathrm{MoS_2}} + \frac{\varepsilon_r \varepsilon_o V_{\mathrm{FB}}}{\mathrm{qd}}$$

Where, ε_o and d are the dielectric constant and thickness of h-BN layer, for the thickness d of 5.5 nm h-BN layer the dielectric constant ε_o is 5.0.

V_{FB} is the flat band voltage caused by the difference between the Fermi levels of graphene (4.7 eV) and MoS_2 (4.2 eV), V_{FB}= 0.5 V.

$$n_{\mathrm{Gr}} = 1.02 \times 10^{17} + \frac{8.85 \times 10^{-12} \times 5 \times 0.5}{1.6 \times 10^{-19} \times 5.5 \times 10^{-9}} = 1.22 \times 10^{17}(\mathrm{m}^{-2}) = 1.22 \times 10^{13}(\mathrm{cm}^{-2})$$

Supplementary Note 4| Comparison performance of TRAM and flash

We directly compared off-current, on-current and on/off ratio of TRAM and Flash memory in the same device (Supplementary Figure 18). We applied silicon back gate for Flash memory operation. Our TRAM shows 2-3 orders lower off-current and 2-3 orders higher on-current than Flash memory, (Supplementary Figure S18c- left panel). These lead TRAM to have an on/off ratio of 10^9 - 5 orders of magnitude higher than flash memory (in same device) and previous reports (on/off ratio of 10^4 in refs. 7, 8) (Supplementary Figure S18c - right panel). Reason is clear. The drain field in TRAM is directly applied on tunneling-oxide that can maximize the charge tunneling (Supplementary Figure 8c), while control gate field in flash memory can reach to tunneling-oxide after pass through blocking-oxide and floating-gate which reduce the actual electric field for charge tunneling (Supplementary Figure 8a).

As mentioned above, control gate field in flash memory becomes weak during passing through blocking-oxide and floating-gate, resulting in high operation voltages. Indeed, previous reports used 15 ~ 18 V for gate pulses to operate the Flash memory. Meanwhile, our TRAM needs low operation voltages of 6 V (3 times smaller) due to the direct application of drain pulses on tunneling-oxide.

Moreover, our TRAM achieved high stretchability over 19% strain due to absence of thick and rigid blocking-oxide and control-gate electrode (Figure 7), while three terminal memory cannot demonstrate stretchability due to the rigid and thick blocking oxide.

Supplementary References

1. Cappelletti, P., Golla, C., Olivo, P., Zanoni, E., Flash Memories, *Springer*, 1st edn (1999).

2. Shklyarevskii, I. N. *et al*. Separation of the contribution of free and bound electrons into real and imaginary parts of the dielectric constant of gold. *USSR. Optika i Spektroskopiya*. **34**, 163 (1973).

3. Salmani-Jelodar, M. *et al.* Single layer MoS_2 band structure and transport. *2011 Int. Semicond. Device Res. Symp.* **6**, 1-2 (2011).

4. Kim, S. M. *et al.* Synthesis of large-area multilayer hexagonal boron nitride for high material performance. *Nat. Commun.* **6,** 8662 (2015).

5. Hwang, C. *et al.* Fermi velocity engineering in graphene by substrate modification. *Sci. Rep.* **2**, 2-5 (2012).

6. Elias, D. C. *et al.* Dirac cones reshaped by interaction effects in suspended graphene. *Nat. Phys.* **7**, 701-704 (2011).

7. Choi, M. S. *et al.* Controlled charge trapping by molybdenum disulphide and graphene in ultrathin heterostructured memory devices. *Nat. Commun.* **4**, 1624 (2013).

8. Simone, B., Daria K., Andras K., Nonvolatile memory cell based on MoS2/graphene heterostructures. *ACS Nano*. **7** (4), 3246–3252 (2013).
